# Food Intake and Physical Activity Patterns Among University Undergraduate Students at Risk of Eating Disorders

**DOI:** 10.3390/nu18010155

**Published:** 2026-01-02

**Authors:** Maria Antònia Amengual-Llofriu, Antoni Aguiló, Pedro Tauler

**Affiliations:** 1 Research Group on Evidence, Lifestyles and Health, Department of Nursing and Physiotherapy, Research Institute of Health Sciences (IUNICS), University of the Balearic Islands, Crta de Valldemossa, km 7.5, 07122 Palma, Balearic Islands, Spain; mariaantonia.amengual@hsll.es; 2 Son Llàtzer University Hospital, 07198 Palma, Balearic Islands, Spain; 3 Health Research Institute of the Balearic Islands (IdISBa), University Hospital Son Espases, Crta de Valldemossa, 79, 07120 Palma, Balearic Islands, Spain; 4 Research Group on Evidence, Lifestyles and Health, Department of Fundamental Biology and Health Sciences, Research Institute of Health Sciences (IUNICS), University of the Balearic Islands, Crta de Valldemossa, km 7.5, 07122 Palma, Balearic Islands, Spain

**Keywords:** eating disorders, physical activity, diet quality, food groups, university undergraduate students

## Abstract

**Background/Objectives**: University students are particularly vulnerable to unhealthy eating patterns and body image dissatisfaction. The association between lifestyle factors and eating disorders (EDs) can be ambiguous as healthier lifestyle choices may paradoxically be related to ED risk. In this study, we aimed to analyze physical activity (PA) and dietary patterns—specifically food type and diet quality—as lifestyle indicators in university students with and without ED risk. Motivations for engaging in PA and the association between PA levels and diet quality were also examined. **Methods**: A descriptive cross-sectional study was conducted on a convenience sample of 1982 undergraduate students aged 18–30 years from the University of the Balearic Islands. Dietary intake, diet quality, PA levels, and motivations were self-reported using a questionnaire. **Results**: Students at risk of EDs reported higher diet quality, including greater adherence to the Mediterranean diet (*p* < 0.001) and more adequate consumption of fruits (*p* < 0.001), vegetables (*p* < 0.001), and red and processed meat (*p* < 0.001). Regarding PA, participants with ED risk engaged in more weekly PA sessions (*p* < 0.001) and accumulated a longer total weekly duration (*p* = 0.019), with physical appearance being the main motivation. In participants without ED risk, PA levels were positively associated with adherence to the Mediterranean diet (*p* < 0.001); however, no such association was observed in participants with ED risk (*p* = 0.538). **Conclusions**: Students at risk for EDs exhibited comparatively healthier diet and PA patterns, seemingly driven by concerns related to body image and an aversion to energy-dense foods. Therefore, apparent health behaviors should not be used to rule out ED risk.

## 1. Introduction

Eating disorders (EDs) and disordered eating behaviors are significant public health concerns, particularly among young adults and university students [1]. The transition from adolescence to adulthood is characterized by greater independence, exposure to new social environments, and increased academic demands, all of which may influence lifestyle behaviors and psychological well-being [2]. University students are particularly vulnerable to developing unhealthy eating patterns, body image dissatisfaction, and maladaptive coping strategies, which may predispose them to EDs or subclinical disordered eating behaviors [3].

Healthy lifestyle choices, characterized by balanced nutrition and regular physical activity (PA), represent well-established protective factors against metabolic disorders, obesity, and psychological distress [4]. High diet quality—defined by an adequate intake of fruits, vegetables, whole grains, and lean proteins, alongside a limited consumption of processed and energy-dense foods—has been associated with improved physical health and better mental health outcomes [5,6,7]. It is noteworthy that these healthier dietary patterns align with those typically reported among individuals with eating disorders, particularly regarding the avoidance of energy-dense foods. However, differences in diet quality between individuals at risk of EDs and those without risk have been scarcely examined. Similarly, engaging in recommended levels of PA is associated with numerous health benefits, including reduced stress, anxiety, and depressive symptoms, as well as improved body image and self-esteem [8]. Therefore, higher levels of PA could be expected in individuals at risk of EDs, who have been shown to exhibit greater concern for body image [9]. However, findings in the literature are contradictory. While some studies have reported higher PA levels among participants with EDs [10], others have failed to demonstrate such differences [11,12].

Importantly, behaviors commonly classified as “healthy”, such as high diet quality or elevated levels of physical activity, may not necessarily reflect a health-driven lifestyle in individuals at risk of eating disorders, as these behaviors can instead be motivated by weight control, body image concerns, or restrictive eating tendencies [1]. In this regard, examining motivations for PA may contribute to a better understanding of PA engagement in this population. Previous studies in university students have reported gender differences in PA motivations [13,14,15,16], and intrinsic motivation has been linked to the adoption of physical activity as part of a balanced and health-oriented lifestyle [15]. Therefore, exploring PA motives not only by gender but also according to ED risk status is essential in understanding how different motivational profiles might contribute to maintaining regular and sustainable PA habits.

Evidence from adolescent and university populations indicates that the combination of regular PA and greater adherence to the Mediterranean diet is associated with enhanced self-esteem, emotional well-being, and a healthier body composition [17]. These outcomes highlight the importance of examining the interaction between PA and dietary behaviors, particularly in populations that may be more vulnerable to body image concerns and disordered eating patterns [17]. Notably, several studies conducted in university students have reported a positive association between higher diet quality and greater PA levels [18,19,20]. However, to the best of our knowledge, this association has not yet been examined in individuals at risk of EDs.

The present study was designed to analyze PA levels and dietary patterns—specifically food types and diet quality—as lifestyle markers in university students with and without risk of EDs. Additionally, motivations for engaging in PA and the association between diet quality and PA levels were examined. We hypothesized that students at risk of EDs would present with higher levels of PA driven primarily by appearance-related motivations. It was also hypothesized that students at risk of EDs would have a more adequate food group intake profile.

## 2. Materials and Methods

### 2.1. Study Design and Participants

A descriptive cross-sectional study was performed on a convenience sample of 1982 undergraduate university students aged 18–30 years old from the University of the Balearic Islands between February and May 2018. The study procedures and participant characteristics have been described in detail elsewhere [21].

Briefly, undergraduate students (18–30 years old) attending their lectures were invited to participate in the study in a voluntary and anonymous fashion. Initially, 2155 students completed the survey. However, 149 respondents (6.9%) were excluded due to incomplete records (n = 125) or because they were outside the set age range (n = 101). This led to a final number of 1982 participants (19.1% of all potential participants), comprising 1278 women (64.5%) and 704 men (35.5%).

All participants were informed of the purpose and requirements of the study. Consent to participate in the study was granted through their agreeing to complete the questionnaire. The study protocol was in agreement with the Declaration of Helsinki for research involving human subjects and was approved by the University of the Balearic Islands Research Ethics Committee (21CER18).

### 2.2. Data Collection

The following self-reported data were collected from participants using a pen-and-pencil questionnaire:

1. The validated Spanish version of the Eating Attitudes Test—26 Items (EAT-26) was used to determine ED risk [22]. The cut-off score to determine ED risk was set at 20 points, with higher scores revealing a risk for EDs [23].

2. Diet quality was determined using an assessment of adherence to the Mediterranean diet. The adherence to the Mediterranean diet was determined using the 14-item questionnaire previously developed and validated for the Spanish population [24]. Each item is scored as 0 or 1, and a global score of 9 or higher indicates good adherence to the Mediterranean diet. Additional questions were included to ascertain the consumption of specific food groups (servings per day or servings per week). The intake of food groups was compared with current Spanish recommendations [25,26]. In addition, the specific meals consumed by the participants each day were identified using the question “Which of the following meals do you consume daily?” The options comprised breakfast, mid-morning snack, lunch, afternoon snack, dinner, and late-night snack.

3. The standard short form of the validated International Physical Activity Questionnaire (IPAQ) provided quantitative information on physical activity levels in metabolic equivalents (MET-h/week). Total weekly physical activity time and daily sitting time were also determined. Furthermore, participants were asked whether they engaged in physical activity during their leisure time. Those who responded affirmatively were subsequently asked about their reasons for engaging in physical activity through a multiple-choice question, for which they could select more than one option. The options provided were as follows: competition, self-improvement, fitness, appearance and body image, social motives, peer influence, fun, stress relief, and health benefits.

### 2.3. Statistical Analysis

Statistical analyses were conducted using IBM SPSS Statistics version 23.0 (IBM Corp., Armonk, NY, USA). A *p* value of less than 0.05 was considered indicative of statistical significance. The normality of the data distribution was assessed using the Kolmogorov–Smirnov test. Descriptive statistics were used to summarize categorical variables as frequencies and percentages, while quantitative variables were reported as medians and interquartile ranges (IQRs, Q25–Q75). Differences between genders and between participants with and without risk of EDs were analyzed using the Mann–Whitney U test or Pearson’s chi-squared (χ^2^) test, as appropriate. When significant differences were detected but interquartile ranges were equal, the average range was applied to identify the higher values. For Mann–Whitney U tests, the effect size was calculated using Rosenthal’s r (low effect: 0.1; median effect: 0.3). For chi-squared analyses, odds ratios (ORs) with 95% confidence intervals or Cramer’s V were computed to estimate the magnitude of the associations. Logistic regression analysis was applied to determine the association between ED risk (YES/NO) and independent variables (adherence to Mediterranean diet and PA levels), with BMI, age, and gender as control variables.

## 3. Results

### 3.1. Characteristics of Participants

The median age of participants was 20 years (range, 19–22). Regarding academic fields, participants were distributed as follows: Social and Legal Sciences, 39.3%; Health Sciences, 25.3%; Engineering and Architecture, 13.9%; Sciences, 13.7%; and Arts and Humanities, 7.7%. Based on BMI classification, 9.4% of participants were underweight, 72.9% had a normal weight, 14.5% were overweight, and 3.2% were classified as obese. The overall risk of EDs was 6.2%, with a higher prevalence in women compared to men (7.9% vs. 3.0%, *p* < 0.001).

### 3.2. Diet Quality and Pattern of Food Intake

Table 1 presents diet quality parameters and intake of selected food groups. Adherence to the Mediterranean diet was higher among women compared with men (38.3% vs. 33.4%, *p* = 0.032, OR: 1.240 (1.019–1.509)), which was reflected in a significantly higher diet score in women (*p* < 0.001). Additionally, women reported a higher number of daily meals (*p* = 0.021).

Regarding food group intake, women consumed more vegetables (*p* < 0.001) and whole-grain foods (*p* = 0.003). In contrast, men reported a higher consumption of red and processed meat (*p* < 0.001), non-red meat (*p* < 0.001), eggs (*p* = 0.003), processed and industrial pastries (*p* = 0.011), potatoes (*p* = 0.007), and refined-grain foods (*p* = 0.004).

**Figure 1:**
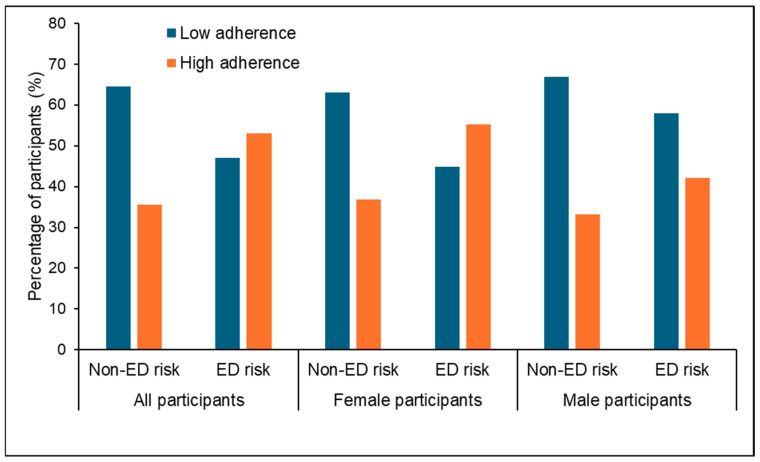
Adherence to the Mediterranean diet in participants with and without ED risk. Values are expressed as the number of participants (percentage). *p* < 0.001 for all participants and for female participants and *p* = 0.413 for male participants as determined by Pearson’s chi-squared test (χ^2^). ED: eating disorder.

Participants at risk of EDs reported a higher adherence to the Mediterranean diet than those without risk (53.0% vs. 35.5%, *p* < 0.001, OR: 2.053 (1.406–2.999); see Figure 1).

Consistently, the Mediterranean diet score was also higher in participants with ED risk (*p* < 0.001; Table 2). These differences remained significant when the analysis was restricted to women (Supplementary Material, Table S1). However, no differences were observed in the number of daily meals between groups.

Table 2 also displays the intake of selected food groups stratified by ED risk status. Participants without ED risk reported a higher consumption of olive oil (*p* = 0.017), butter and cream (*p* < 0.001), red and processed meat (*p* < 0.001), refined-grain cereals (*p* < 0.001), and processed and industrial pastries (*p* < 0.001). Conversely, participants with ED risk consumed more vegetables (*p* < 0.001), fruits (*p* < 0.001), and whole-grain foods (*p* < 0.001). A similar pattern was observed when analyses were performed separately for male and female participants (Supplementary Material, Table S1) although statistical significance for both sexes was only maintained for butter and cream, refined-grain cereals, and processed and industrial pastries.

**Figure 2:**
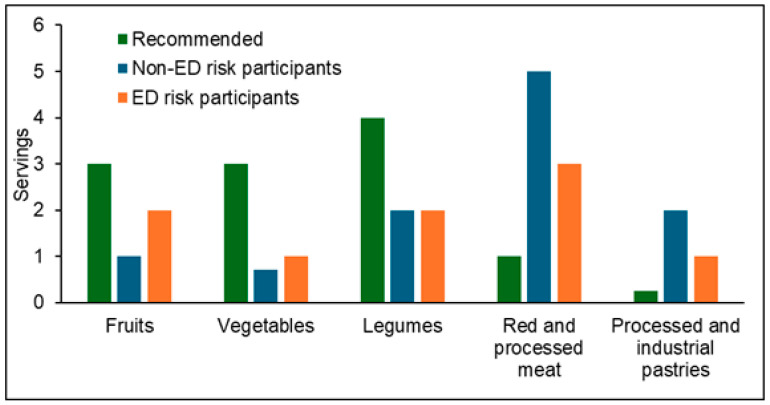
Intake of selected food groups compared to recommended values in participants with and without risk of EDs. Values are expressed as servings/day (fruits and vegetables) or servings/week. The following recommendations were considered: fruit, 3 servings/day; vegetables, 3 servings/day; legumes, 4 servings/week; red and processed meat, 1 serving/week (occasional consumption); processed and industrial pastries, 0.25 serving/week (1 serving/month, sporadic consumption). ED: eating disorder.

When the intake of specific food groups was compared with current Spanish dietary recommendations, participants at risk of EDs reported a more adequate consumption profile overall. However, an excessive intake of red and processed meat and an insufficient intake of fruits, vegetables, and legumes were observed in both groups (Figure 2).

### 3.3. Physical Activity Levels and Motivations

Table 3 presents the PA levels among participants stratified by gender. A greater proportion of men were classified within the vigorous PA level, whereas a higher proportion of women fell into the moderate and low PA categories (*p* < 0.001, Cramer’s V = 0.3). Overall, men reported significantly higher values for total and vigorous physical activity parameters. No significant differences were observed between genders in moderate PA, walking, and sitting time. In addition, a higher percentage of men reported engaging in leisure-time PA (*p* < 0.001, OR: 0.510 (0.418–0.622)).

Table 4 presents the motivations for engaging in PA during leisure time among participants, stratified by gender. When these motivations were analyzed, significant gender differences were observed for all motivations except for fitness (*p* = 0.762). Motivations such as competition (*p* < 0.001), self-improvement (*p* < 0.001), social interaction (*p* < 0.001), peer influence (*p* < 0.001), and fun (*p* < 0.001) were more prevalent among men. In contrast, motivations related to appearance and body image (*p* < 0.001), stress relief (*p* < 0.001), and health benefits (*p* < 0.001) were more prevalent in women. Across all participants, the most frequently reported motivations were stress relief (80.0%), fitness (75.5%), and health benefits (74.5%).

**Figure 3:**
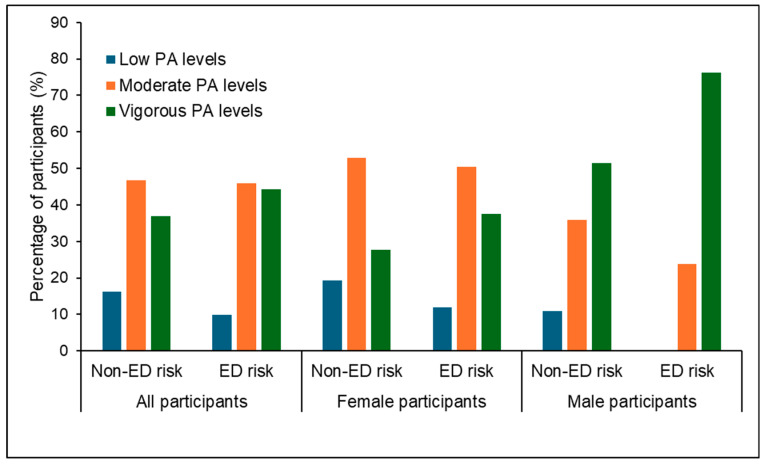
Physical activity levels in participants with and without eating disorder risk. Values are expressed as the number of participants (percentage). *p* = 0.100 for all participants, *p* = 0.049 for female participants, and *p* = 0.074 for male participants as determined using Pearson’s chi-squared test (χ^2^). No male participants with ED risk reported low PA levels. ED: eating disorder.

Figure 3 shows the distribution of participants across PA levels, stratified by ED risk. A slightly higher proportion of women at risk for EDs were classified into the vigorous PA category (37.6% vs. 27.8%, *p* = 0.049. Cramer’s V = 0.1).

Table 5 presents the PA parameters among participants, stratified by ED risk. Overall, participants at risk for EDs engaged in more weekly PA sessions (*p* < 0.001) and accumulated a longer total weekly duration of PA (*p* = 0.019). Across the entire sample—and particularly when stratified by gender (Supplementary Material, Table S2)—most of the differences between participants with and without ED risk were observed in the vigorous and moderate PA parameters, with higher values among those at risk. Moreover, differences were more marked in the frequency of weekly sessions than in the duration of individual sessions. No significant differences were found in walking-related PA parameters. Additionally, a higher percentage of participants at risk of EDs reported engaging in leisure-time PA (73.1% vs. 62.1%, *p* = 0.042, OR: 1.517 (1.014–2.271)).

Table 6 shows the PA motivations among participants stratified by ED risk. Participants at risk of EDs were significantly more likely to report exercising for appearance and body image reasons (92.7% vs. 59.2%, *p* < 0.001). This was the most prevalent motivation among participants with ED risk and represented the highest proportion observed across all motivation categories in the study. Conversely, a significantly lower percentage of participants at risk of EDs reported exercising for fun (43.9% vs. 70.9%, *p* < 0.001). Appearance and body image was the only motivation showing significant differences in both genders (Supplementary Material, Table S3).

### 3.4. Multivariate Logistic Regression Analysis for ED Risk

Table 7 shows the results of the logistic regression analysis for ED risk. Participants with ED risk were more likely to be female (*p* < 0.001) and obese (*p* = 0.006) with vigorous PA levels (*p* = 0.031) and a high adherence to the Mediterranean diet (*p* = 0.002).

### 3.5. Association Between PA Level and Adherence to the Mediterranean Diet

**Figure 4:**
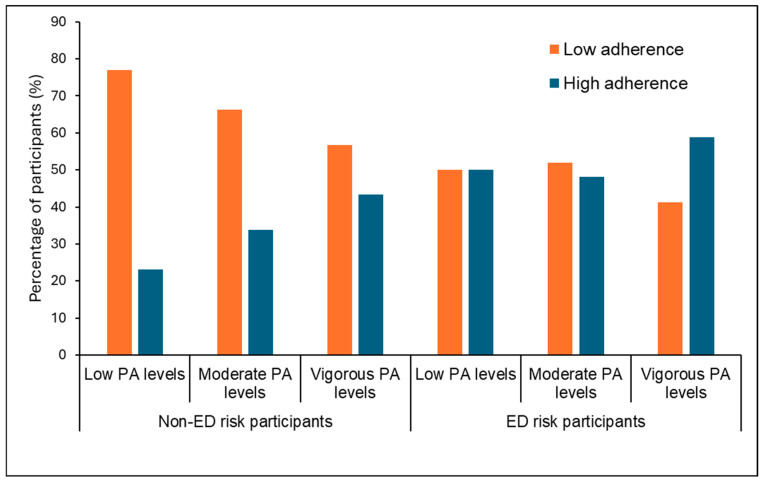
Association of adherence to the Mediterranean diet and physical activity levels in participants with and without eating disorder risk. Values are expressed as the number of participants (percentage). *p* < 0.001 for non-ED-risk participants (Cramer’s V = 0.1) and *p* = 0.538 for participants with ED risk as determined using Pearson’s chi-squared test (χ^2^). ED: eating disorder, PA: physical activity.

Figure 4 illustrates the association between PA levels and adherence to the Mediterranean diet in participants stratified by ED risk. Among participants without ED risk, PA levels were significantly associated with Mediterranean diet adherence (*p* < 0.001), with higher adherence observed in those with higher PA levels. In contrast, no such association was found among participants at risk of EDs (*p* = 0.538).

## 4. Discussion

The main findings of the present study indicate that participants at risk of EDs reported higher diet quality compared with participants without ED risk. This higher diet quality was reflected in an increased consumption of fruits and vegetables and a reduced intake of high-fat foods. Regarding PA, individuals at risk of EDs also reported higher PA levels, with appearance-related concerns representing the primary motivator for engaging in exercise. Notably, the association typically observed between higher PA levels and greater adherence to the Mediterranean diet was not found among participants at risk of EDs.

Our study revealed gender-based differences in several diet quality parameters. For example, female participants reported a higher adherence to the Mediterranean diet, a greater number of daily meals, and a more adequate intake of food groups such as vegetables, red and processed meat, whole-grain and refined cereals, and processed and industrial pastries. The main differences observed between genders are consistent with previous findings [20,27]. It has been suggested that, because women tend to be more concerned about maintaining their weight, they pay greater attention to appropriate food intake [9,20]. Furthermore, the higher motivation for physical activity observed in the present study among women for health-related reasons may also contribute to this improved dietary pattern. However, despite these differences, it should be noted that both genders demonstrated a low adherence to the Mediterranean diet, as well as an inadequate intake of key food groups such as fruits, vegetables, and cereals, which is consistent with previous reports [28,29].

Significant differences were also observed when food intake was analyzed in participants at risk of EDs and in those without ED risk, with individuals at risk demonstrating a more adequate dietary pattern and, consequently, greater adherence to the Mediterranean diet. This association between ED risk and higher adherence to the Mediterranean diet has rarely been reported, as only one previous study has described a relationship between lower Mediterranean diet adherence and higher anxiety dimension scores [30]. Differences in participant characteristics—such as higher BMI values [31], older age (non-students) [32], or even the use of different tools to assess adherence [32]—may account for these discrepancies. The higher adherence to the Mediterranean diet observed in the present study is consistent with a more favorable dietary profile, characterized by a lower consumption of butter and cream, red and processed meat, refined grains, and processed and industrial pastries, alongside higher intakes of fruits and vegetables [33]—all core components used to evaluate Mediterranean diet adherence. These differences suggest a healthier dietary pattern among participants at risk of EDs, mainly through reduced fat intake but also by restricting the consumption of processed and sugar-rich foods [34]. From a theoretical perspective, this paradox may be explained by established models of eating disorder psychopathology in which higher diet quality reflects cognitive dietary restraint, the internalization of body ideals, and the moralization of “healthy” eating rather than a flexible and genuinely health-oriented dietary pattern [32,33]. However, it is notable that, regarding one of the most characteristic elements of the Mediterranean diet—olive oil consumption—our study found that individuals at risk of EDs consumed lower amounts of this type of oil. This observation is consistent with findings from the SUN study, which reported an inverse association between olive oil consumption and the risk of anorexia and bulimia nervosa [32]. As previously suggested, this may be explained by attempts to avoid any sources of fat or energy-dense foods, which is further supported by the reduced intake of processed and industrial pastries observed among participants at risk of EDs. Therefore, similarly to what was observed in women, individuals at risk of EDs reported stronger concern for weight control and body image and health-related motivations, which coincides with these improved dietary patterns.

In agreement with previous findings [10], the results of the present study revealed that, overall, participants at risk of EDs reported higher levels of physical activity. However, it is noteworthy that these differences were mainly observed in parameters related to moderate and vigorous physical activity, while no differences were detected in walking-related variables. This finding has not been reported frequently; in fact, it has even been suggested that walking is the most common form of exercise among individuals at risk of EDs due to its accessibility [35,36]. Individuals at risk of EDs may engage in intense physical activity as a means of emotional regulation or as a compensatory behavior rather than purely for health-related purposes. Physical activity triggers the release of endorphins and serotonin, temporarily alleviating anxiety, guilt, or sadness, thereby reinforcing its role as an emotional coping mechanism. Moreover, perceived control over one’s body and the social approval associated with “healthy” exercise behaviors may further strengthen this association. Consequently, physical activity may serve as a socially acceptable strategy for managing emotional distress in vulnerable individuals [37]. In addition, traits such as perfectionism, rigidity, and the internalization of body ideals may contribute to more intense training sessions and reduced recovery time between them. Consistent with this observation, participants at risk of EDs tended to complete a higher number of weekly physical activity sessions. Complementary to this, the finding that these individuals engaged in more frequent sessions of varying intensities yet did not consistently achieve a higher overall volume or duration of physical activity may suggest that the consistency of physical activity engagement—rather than its total quantity—may indicate greater vulnerability.

Although students at risk of EDs exhibited dietary and physical activity patterns that superficially resemble health-promoting behaviors, these patterns may reflect rigid control, pathological restraint, or compulsive exercise rather than adaptive lifestyle choices [34,38]. Such behaviors should be interpreted with caution, particularly in light of evidence linking orthorexic tendencies and appearance-driven exercise with eating disorder psychopathology [34,38]. From a public health perspective, this paradox warrants particular attention. Although healthier dietary behaviors and higher levels of physical activity are generally considered protective, in individuals at risk of EDs, these behaviors may conceal underlying pathology and complicate early identification.

In agreement with these points, individuals at risk of EDs appeared to engage in physical activity primarily due to concerns about appearance and body image, whereas fun or enjoyment represented a less prominent motivation compared to those without ED risk [39,40,41]. Although the effect sizes and association magnitudes observed in the present study were small overall, the association showing that individuals at risk of EDs engaged in physical activity mainly due to appearance- and body image-related concerns stood out as the strongest among the observed relationships, with a clear difference compared to the others. From the perspective of Self-Determination Theory, appearance- and body image-driven motives can be conceptualized as more controlled forms of motivation, whereas motives related to enjoyment or intrinsic interest reflect more autonomous regulation. In this context, the predominance of appearance-related motivation among participants at risk of EDs suggests a more controlled regulatory style, which has been associated with less adaptive exercise patterns, including compulsive or rigid physical activity behaviors and poorer psychological outcomes [42]. Conversely, the greater endorsement of enjoyment-related motives among participants without ED risk is consistent with more autonomous motivation, which has been linked to greater psychological well-being and more sustainable engagement in physical activity [43].

Regarding the analysis of the motivations behind PA in men and women, the present study revealed distinct gender-related patterns: social motivations (such as social interaction and peer influence), enjoyment, performance, and competitive motives were more prevalent among men, whereas appearance-related motives, health, and stress relief were more common among women. These findings are consistent with previous research conducted among university students, indicating that men are more motivated by social, performance, and competitive factors, while women are more driven by concerns related to appearance, weight management, health, and stress relief [13,14,15,16]. Gender differences in aspects such as a higher concern for body image [9] or higher stress levels reported by female university students [44,45] may help explain some of the motivational differences observed, not only between genders but also between participants with and without ED risk. In this regard, it has been commonly suggested that men tend to exhibit higher levels of intrinsic motivation, whereas women may show more extrinsic or partially internalized motivation, making them potentially more vulnerable to declines in motivation if appearance-related pressures or social comparison dynamics change [13,14,15,16]. Indeed, intrinsic motivation has been associated with higher levels of physical activity and more stable exercise behavior [15], which aligns with the higher physical activity levels observed among male university students in the present study, as well as in previous research [16].

The findings of our study revealed a positive association between higher levels of physical activity and better diet quality [18,19,20]. This observation is consistent with previous evidence suggesting that healthy behaviors tend to cluster, particularly those related to physical activity and dietary habits, as previously reported by our group in university students [27]. Interestingly, in the present study, this association was not observed among participants with ED risk. This finding suggests that ED risk may act as a moderating factor, potentially influencing or attenuating the relationship between physical activity and diet quality. A possible explanation for the different patterns observed between individuals with and without ED risk may lie in the underlying motivations for engaging in physical activity. For instance, individuals at risk of EDs may participate in physical activity primarily for appearance-related reasons rather than health-related goals, which could influence both their dietary choices and the strength of the association between physical activity and diet quality. Furthermore, the generally higher adherence to the Mediterranean diet observed among participants with ED risk may have contributed to the absence of a clear association between physical activity and diet quality in this subgroup.

This study has several limitations that should be acknowledged. Given the cross-sectional observational design, causal inferences cannot be drawn. The temporal ordering of the variables assessed cannot be established, and observed associations may reflect reverse causation or bidirectional relationships. Therefore, the findings should be interpreted as descriptive and associative rather than indicative of causal mechanisms. All variables were assessed using self-reported measures, which may be subject to recall bias and social desirability effects, potentially leading to the misreporting or underreporting of certain behaviors or symptoms. The study relied on data collected from students at a single university, with a limited response rate, which may limit the generalizability of the findings. Given the number of statistical tests conducted, the risk of type I error cannot be ruled out. Furthermore, the prevalence of ED risk was relatively low, which may have reduced the statistical power of some stratified analyses, potentially preventing the detection of significant associations. In particular, the low prevalence of ED risk among men in the sample resulted in small subgroup sizes, which may have limited the statistical power and the stability of estimates for this group. Therefore, findings related to sex-specific comparisons should be interpreted with caution. An additional limitation is the use of a single EAT-26 cutoff to define ED risk without distinguishing between different ED symptom profiles. This aggregation may obscure potentially distinct patterns due to differences across characteristics of ED types. Consequently, subtype-specific differences could not be examined in the present study.

## 5. Conclusions

Within an overall inadequate dietary profile, participants at risk of EDs displayed a comparatively healthier pattern, although this appeared to be associated with their aversion to consuming high-energy foods. Regarding physical activity, participants with ED risk generally showed slightly higher activity levels, with physical appearance being their main motivation. It is important to note that, while a clear association between diet quality and physical activity levels was observed among participants without ED risk, such an association was not found in those with EDs.

These findings suggest that interventions targeting diet and physical activity should consider these differences as approaches that are effective for individuals without ED risk may not be appropriate for those with it. Importantly, the presence of apparently “healthy” lifestyle behaviors should not be taken as evidence that ED risk is absent, highlighting the need for careful screening and assessment. From a public health perspective, this paradox warrants particular attention. Although healthier dietary behaviors and higher levels of physical activity are generally considered protective, in individuals at risk of eating disorders, these behaviors may conceal underlying pathology and complicate early identification.

## Figures and Tables

**Table 1:** Diet quality and food intake of participants in the study.

	All (*n* = 1982)	Women (*n* = 1278)	Men (*n* = 704)	*p* Value (r)
**Adherence to Mediterranean diet**				0.032 * (0.1)
Low	1210 (63.5)	755 (61.7)	455 (66.6)	
High	697 (36.5)	469 (38.3)	228 (33.4)	
Score	8 (7–9)	8 (7–9)	8 (6–9)	<0.001 * (0.1)
**Number of daily meals**	4 (3–5)	4 (4–5)	4 (3–5)	0.021 * (0.1)
**Food intake** (servings·day^−1^)				
Olive oil	2 (2–3)	3 (2–3.5)	2 (2–3)	0.176
Vegetables	0.7 (0.4–1.4)	0.7 (0.4–1.5)	0.71 (0.43–1.0)	<0.001 * (0.1)
Fruits	1 (0.6–2)	1 (0.57–2)	1 (0.6–2)	0.052
Dairy products	1 (1–2)	1 (1–2)	1 (1–2)	0.115
Whole-grain cereals	0.43 (0.13–1)	0.43 (0.14–1)	0.29 (0–0.86)	0.003 * (0.1)
Refined cereals	0.43 (0.29–0.86)	0.43 (0.29–0.71)	0.43 (0.29–1)	0.004 * (0.1)
Potatoes	0.4 (0.3–0.7)	0.43 (0.29–0.71)	0.43 (0.29–0.71)	0.007 * (0.1)
**Food intake** (servings·week^−1^)				
Red/processed meat	5 (3–7)	4 (2–7)	7 (4–11)	<0.001 * (0.1)
Non-red meat	3 (2–5)	3 (2–5)	3 (2–5)	<0.001 * (0.1)
Fish and seafoods	2 (1–2.5)	2 (1–2.5)	2 (1–2)	0.629
Butter and cream	1 (0–2)	1 (0–2)	1 (0–2)	0.189
Legumes	2 (1–2)	2 (1–2)	2 (1–2)	0.663
Eggs	2 (1–3)	2 (1–3)	2 (2–4)	0.003 * (0.1)
Nuts	2 (1–4)	2 (1–3)	2 (1–4)	0.663
Processed/Industrial pastries	2 (1–4)	2 (1–4)	2 (1–4)	0.011 * (0.1)

Values are expressed as the median (25th–75th percentile) or number of participants (percentage). * *p* < 0.05 indicates significant differences between men and women as determined by the Mann–Whitney U test or Pearson’s chi-squared test (χ^2^).

**Table 2:** Diet quality and food intake of participants stratified by risk of eating disorders.

	No ED Risk (n = 1883)	ED Risk (n = 123)	*p* Value (r)
**Adherence to Mediterranean diet** (score)	8 (7–9)	9 (7–10)	<0.001 * (0.1)
**Number of daily meals**	4 (3–5)	4 (3–5)	0.995
**Food intake** (servings·day^−1^)			
Olive oil	2.5 (2.0–3.5)	2 (1–3)	0.017 * (0.1)
Vegetables	0.71 (0.43–1.14)	1 (0.57–2)	<0.001 * (0.1)
Fruits	1.0 (0.57–2.0)	2 (1–3)	<0.001 * (0.1)
Dairy products	1 (1–2)	1 (0.57–2)	0.175
Whole grains	0.43 (0–1)	0.43 (0.29–1)	0.005 * (0.1)
Refined cereals	0.43 (0.29–0.86)	0.29 (0.14–0.71)	<0.001 * (0.1)
Potatoes	0.43 (0.29–0.71)	0.43 (0.29–0.71)	0.039 * (0.1)
**Food intake** (servings·week^−1^)			
Red/processed meat	5 (3–7)	3 (2–7)	<0.001 * (0.1)
Non-red meat	3 (2–5)	3.5 (2–6.63)	0.325
Fish and seafoods	1 (1–2)	2 (1–3)	0.332
Butter and cream	1 (0–2)	0 (0–1)	<0.001 * (0.1)
Legumes	2 (1–2)	2 (1–3)	0.946
Eggs	2 (1–3)	2 (1–4.75)	0.275
Nuts	2 (1–4)	2 (1–4)	0.600
Processed/Industrial pastries	2 (1–4)	1 (0–2)	<0.001 * (0.1)

Values are expressed as the median (25th–75th percentile). * *p* < 0.05 indicates significant differences between participants at risk and participants with no risk as determined by the Mann–Whitney U test.

**Table 3:** Physical activity levels of study participants.

	All (*n* = 1982)	Women (*n* = 1278)	Men (*n* = 704)	*p* Value (r)
**IPAQ classification**				<0.001 *
Low (%)	315 (15.9)	240 (18.8)	75 (10.7)	
Moderate (%)	924 (46.6)	673 (52.7)	251 (35.7)	
Vigorous (%)	743 (37.5)	365 (28.5)	378 (53.7)	
**Total PA**				
MET-h/week	25.0 (11.3–46.5)	21.0 (10.3–38.5)	37.3 (18.2–61.1)	<0.001 * (0.3)
Sessions/week	9.0 (7.0–12.0)	8.0 (6.0–11.0)	10.0 (7.0–13.0)	<0.001 * (0.2)
Minutes/week	410.0 (220.0–655.0)	360.0 (210.0–581.3)	475.0 (280.0–750.0)	<0.001 * (0.2)
**Intense PA**				
Sessions/week	2.0 (0–3.0)	1.0 (0–3.0)	3.0 (1.0–4.0)	<0.001 * (0.3)
Minutes/day	30.0 (0–60.0)	10.0 (0–60.0)	60.0 (11.2–90.0)	<0.001 * (0.3)
Minutes/week	60.0 (0–225.0)	10.0 (0–150.0)	160.0 (21.2–360.0)	<0.001 * (0.3)
**Moderate PA**				
Sessions/week	2.0 (0–3.0)	2.0 (0–3.0)	2.0 (0–3.0)	0.328
Minutes/day	30.0 (0–60.0)	34.0 (0–62.0)	30.0 (0–60.0)	0.457
Minutes/week	60.0 (0–175.0)	60.0 (0–160.0)	60.0 (0–180.0)	0.492
**Walking**				
Sessions/week	6.0 (4.0–7.0)	6.0 (4.0–7.0)	6.0 (4.0–7.0)	0.536
Minutes/day	30.0 (20.0–60.0)	30.0 (20.0–60.0)	30.0 (20.0–60.0)	0.051
Minutes/week	157.0 (90.0–315.0)	175.0 (90.0–315.0)	150.0 (90.0–300.0)	0.426
**Sitting time** (hours/day)	8.0 (6.0–10.0)	8.0 (6.0–10.0)	8.0 (6.0–10.0)	0.541
**Leisure PA** (yes)	1242 (62.7)	732 (57.3)	510 (72.4)	<0.001 *

Values are expressed as the median (25th–75th percentile) or number of participants (percentage). * *p* < 0.05 indicates significant differences between men and women as determined by the Mann–Whitney U test or Pearson’s chi-squared test (χ^2^). PA: physical activity.

**Table 4:** Physical activity motivations of study participants.

Motivation	All (*n* = 1242)	Women (*n* = 732)	Men (*n* = 510)	OR (95% CI)	*p* Value
Competition	313 (25.2)	95 (13.0)	218 (42.7)	0.198 (0.150–0.263)	<0.001 *
Self-improvement	594 (47.8)	304 (41.6)	290 (56.8)	0.543 (0.430–0.684)	<0.001 *
Fitness	938 (75.5)	551 (75.3)	387 (75.9)		0.762
Appearance and body image	771 (62.1)	490 (66.9)	281 (55.2)	1.614 (1.275–2.043)	<0.001 *
Social interaction	313 (25.2)	155 (21.2)	158 (30.9)	0.595 (0.458–0.774)	<0.001 *
Peer influence	102 (8.2)	37 (5.0)	65 (12.7)	0.356 (0.232–0.547)	<0.001 *
Fun	857 (69.0)	464 (63.2)	393 (77.1)	0.507 (0.391–0.656)	<0.001 *
Stress relief	994 (80.0)	617 (84.2)	377 (73.9)	1.863 (1.403–2.476)	<0.001 *
Health	925 (74.5)	580 (79.3)	345 (67.9)	1.822 (1.403–2.366)	<0.001 *

Values are expressed as the number of participants (percentage). * *p* < 0.05 indicates significant differences between men and women, as determined using Pearson’s chi-squared test (χ^2^). Odds ratios (ORs) with 95% confidence intervals are provided to estimate the magnitude of the significant associations (reference: male).

**Table 5:** Physical activity levels in participants stratified by risk of eating disorders.

		All	
	Non-ED Risk (n = 1860)	ED Risk (n = 122)	*p* Value (r)
**Total PA**			
MET-h/week	24.7 (11.3–46.5)	28.4 (13.1–51.5)	0.152
Sessions/week	9.0 (7.0–12.0)	10.0 (7.0–13.0)	<0.001 * (0.1)
Minutes/week	400.0 (210.0–645.0)	470.0 (270.0–828.8)	0.019 * (0.1)
**Intense PA**			
Sessions/week	1.0 (0–3.0)	2.0 (0–4.0)	0.051
Minutes/session	30.0 (0–60.0)	40.0 (0–60.0)	0.361
Minutes/week	60.0 (0–221.5)	90.0 (27.5–202.5)	0.133
**Moderate PA**			
Sessions/week	2.0 (0–3.0)	3.0 (1.0–4.0)	<0.001 * (0.1)
Minutes/session	30.0 (0–60.0)	32.5 (10.0–60.0)	0.178
Minutes/week	60.0 (0–150.0)	36.0 (13.0–63.6)	0.006 * (0.1)
**Walking**			
Sessions/week	6.0 (4.0–7.0)	6.0 (4.0–7.0)	0.983
Minutes/session	30.0 (20.0–60.0)	40.0 (20.0–60.0)	0.075
Minutes/week	150.0 (90.0–315.0)	200.0 (90.0–366.3)	0.155
**Sitting time** (hours/day)	8.0 (6.0–10.0)	8.0 (6.0–10.0)	0.666
**Leisure PA** (yes)	1155 (62.1)	87 (73.1)	0.042 *

Values are expressed as the median (25th–75th percentile) or number of participants (percentage). * *p* < 0.05 indicates significant differences between participants at risk and participants with no risk, as determined using the Mann–Whitney U test or Pearson’s chi-squared test (χ^2^). r, as a measure of effect size, is provided for significant differences (Mann–Whitney U test). PA: physical activity.

**Table 6:** Physical activity motivations in participants stratified by risk of eating disorders.

	Non-ED Risk (n = 1155)	ED Risk (n = 87)	OR (95% CI)	*p* Value
Competition	298 (25.8)	15 (17.2)		0.079
Self-improvement	551 (47.7)	43 (49.4)		0.685
Fitness	873 (75.6)	65 (74.7)		0.618
Appearance and body image	690 (59.2)	81 (93.1)	8.520 (3.679–19.728)	<0.001 *
Social interaction	293 (25.4)	20 (23.0)		0.482
Peer influence	97 (8.4)	5 (5.8)		0.262
Fun	819 (70.9)	38 (43.7)	0.322 (0.205–0.508)	<0.001 *
Stress relief	920 (79.7)	74 (85.1)		0.204
Health	860 (74.5)	65 (74.7)		0.818

Values are expressed as number of participants (percentage). * *p* < 0.05 indicates significant differences between participants at risk and participants with no risk as determined using Pearson’s chi-squared test (χ^2^). Odds ratios (ORs) with 95% confidence intervals were computed to estimate the magnitude of the significant associations (reference: non-ED risk). ED: eating disorder.

**Table 7:** Logistic regression analysis for ED risk.

Variable	Adjusted OR	95% CI	*p* Value
**Adherence Mediterranean diet** (reference: “low adherence”)	1.837	1.241–2.717	0.002 *
**PA level** (reference: “moderate”)			0.010 *
Low	0.579	0.288–1.164	0.125
Vigorous	1.586	1.042–2.412	0.031 *
**Sex** (reference: “male”)	3.565	2.115–6.008	<0.001 *
**Age**	0.960	0.884–1.044	0.341
**BMI** (reference: “normal weight”)			0.012 *
Underweight	0.589	0.265–1.309	0.194
Overweight	1.379	0.812–2.343	0.234
Obese	3.134	1.384–7.100	0.006 *

Reference: participants with no risk of eating disorders. * *p* < 0.05 indicates significant predictors and odds ratios (ORs). R^2^: 0.078 (*p* < 0.001); PA: physical activity; BMI: body mass index; OR: odds ratio.

## Data Availability

The data presented in this study are available on request from the corresponding author.

## Supplementary Materials

The following supporting information can be downloaded at https://www.mdpi.com/article/10.3390/nu18010155/s1, Table S1. Diet quality and food intake of participants stratified by risk of eating disorders and gender; Table S2. Physical activity levels in participants stratified by gender and by risk of eating disorders; Table S3. Physical activity motivations in participants stratified by gender and by risk of eating disorders.

## Abbreviations

The following abbreviations are used in this manuscript:

ED	Eating disorder
PA	Physical activity
IPAQ	International Physical Activity Questionnaire
IQR	Interquartile range
