# Supplementary material for: Food Intake and Physical Activity Patterns Among University Undergraduate Students at Risk of Eating Disorders
Source: Nutrients. 2026 Jan 2;18(1):155. doi: 10.3390/nu18010155 (PMC12787788; doi:10.3390/nu18010155)
Supplement: Supplementary file 1 [file nutrients-18-00155-s001.zip › nutrients-4052997-supplementary.pdf]

Table S1. Diet quality and food intake of participants stratified by risk of eating disorders and gender

	Women			Men		
	**No-ED risk** (n = 1192)	**ED risk** (n = 102)	**p value (r)**	**No-ED risk** (n = 691)	**ED risk** (n = 21)	**p value (r)**
**Adherence to Mediterranean Diet** (score)	8 (7-9)	9 (7-10)	<0.001* (0.1)	8 (6-9)	8 (6-10)	0.276
**Number of daily meals**	4 (4-5)	4 (3-5)	0.855	4 (3-5)	4 (3-5)	0.852
**Food intake** (servings/day)						
Olive oil	3 (2-4)	2 (1-3)	0.070	2 (2-3)	2 (1-3)	0.034* (0.1)
Vegetables	0.71 (0.43-1.50)	1.43 (0.57-2)	<0.001* (0.1)	0.71 (0.43-1.0)	1 (0.46-1.21)	0.239
Fruits	1.0 (0.57-2.0)	2 (1-3)	<0.001* (0.2)	1 (0.57-2)	1 (0.57-3)	0.576
Dairy products	1 (1-2)	1 (0.57-2)	0.396	1 (1-2)	1 (0.43-2)	0.275
Whole grain	0.43 (0.14-1.0)	0.43 (0.27-1)	0.072	0.29 (0-0.86)	0.57 (0.18-1)	0.235
Refined grain cereals	0.43 (0.29-0.71)	0.27 (0.14-0.59)	<0.001* (0.1)	0.43 (0.29-1)	0.43 (0.40-1)	0.035*(0.1)
Potatoes	0.43 (0.29-0.71)	0.43 (0.29-0.71)	0.088	0.43 (0.29-0.71)	0.43 (0.29-0.79)	0.436
**Food intake** (servings/week)						
Red / processed meat	5 (3-7)	3 (1.13-7)	0.003* (0.1)	7 (4-12)	4 (2.5-7)	0.100
Non-red meat	3 (2-5)	3 (2-6.75)	0.624	3 (2-5)	5 (3-6.5)	0.047
Fish and seafoods	2 (1-2.5)	2 (1-3)	0.697	2 (1-2)	2 (1-3)	0.179
Legumes	2 (1-2)	2 (1-2.63)	0.936	2 (1-2)	2 (0.75-3)	0.922
Butter and cream	1 (0-2)	0 (0-1)	<0.001* (0.1)	1 (0-2)	0	0.003*(0.1)
Nuts	2 (1-2)	2 (0-4)	0.554	2 (1-4)	4 (0-7)	0.383
Eggs	2 (1-3)	2 (1-4)	0.536	2 (2-4)	3 (2-6)	0.083
Processed / Industrial pastries	2 (1-4)	1 (0-2)	<0.001 (0.1)	2 (1-4)	1 (0.2)	0.009*

Values are expressed as median (25th - 75th percentile). * $p < 0.05$ indicates significant differences between participants at risk and participants with no risk, as determined by U-Mann Whitney test. r, as a measure of effect size, is provided for significant differences (Mann–Whitney U test). ED: eating disorder.

Table S2. Physical activity levels in participants stratified per gender and by risk of eating disorders

	Women			Men		
	**No-ED risk** (n = 1177)	**ED risk** (n = 101)	**p value (r)**	**No-ED risk** (n = 683)	**ED risk** (n = 21)	**p value**
**Total PA**						
MET-h/week	21.0 (10.2-38.0)	25.4 (10.9-41.0)	0.127	36.9 (17.9-59.2)	63.9 (37.3-94.1)	<0.001*(0.1)
Sessions/week	8.0 (6.0-10.0)	10.0 (7.0-12.0)	0.004*(0.1)	10.0 (7.0-13.0)	13.0 (10.0-15.0)	<0.001*(0.1)
Minutes/week	360.0 (202.5-575.5)	420.0 (255.0-625.0)	0.074	460.0 (270.0-750.0)	825.0 (585.0-1280.0)	<0.001*(0.1)
**Intense PA**						
Sessions/week	0 (0-3.0)	2.0 (0-3.0)	0.005*(0.1)	3.0 (1.0-4.0)	4.0 (2.0-5.0)	0.030*(0.1)
Minutes/session	0 (0-60.0)	30.0 (0-60.0)	0.057	60.0 (10.0-90.0)	75.0 (60.0-120.0)	0.056
Minutes/week	0 (0-135.0)	60.0 (0-180.0)	0.016*(0.1)	150.0 (20.0-360.0)	300.0 (120.0-550.0)	0.012*(0.1)
**Moderate PA**						
Sessions/week	2.0 (0-3.0)	3.0 (1.0-4.0)	<0.001*(0.1)	2.0 (0-3.0)	2.0 (0.5-4.5)	0.228
Minutes/session	30.0 (0-60.0)	33.0 (13.0-63.0)	0.600	30.0 (0-60.0)	60.0 (7.5-120.0)	0.033*(0.1)
Minutes/week	60.0 (0-150.0)	90.0 (25.0-180.0)	0.025*(0.1)	60 (0-175.0)	120.0 (15.0-430.0)	0.057
**Walking**						
Sessions/week	6.0 (4.0-7.0)	5.0 (4.0-7.0)	0.352	6.0 (4.0-7.0)	7.0 (5.5-7.0)	0.032*(0.1)
Minutes/session	30.0 (20.0-60.0)	30.0 (20.0-60.0)	0.332	30.0 (20.0-60.0)	45.0 (17.5-75.0)	0.095
Minutes/week	180.0 (90.0-340.0)	175.0 (90.0-315.0)	0.575	150.0 (90.0-300.0)	315.0 (97.5-420.0)	0.058
**Sitting time** (hours/day)	8.0 (6.0-10.0)	8.0 (6.0-10.0)	0.666	8.0 (6.0-10.0)	8.0 (4.5-10)	0.760
**Leisure PA (yes)**	663 (56.3)	69 (68.3)	0.019*(0.1)	492 (72.0)	18 (85.7)	0.167

Values are expressed as median (25th - 75th percentile) or number of participants (percentage). * $p < 0.05$ indicates significant differences between participants at risk and participants with no risk, as determined by U-Mann Whitney test or Pearson's chi-square ($\chi2$). r, as a measure of effect size, is provided for significant differences (Mann–Whitney U test). ED: eating disorder, PA: physical activity.

Table S3. Physical activity motivations in participants stratified by gender and by risk of eating disorders

	Women				Men			
	**No-ED risk** (n = 1177)	**ED risk** (n = 101)	**OR (95%CI)**	**p Value**	**No-ED risk** (n = 683)	**ED risk** (n = 21)	**OR (95%CI)**	**p value**
Competition	88 (13.3)	7 (10.1)		0.351	210 (42.4)	8 (44.4)		0.703
Self-improvement	274 (41.3)	30 (43.5)		0.607	277 (56.3)	13 (70.6)		0.237
Fitness	500 (75.4)	51 (73.9)		0.579	373 (75.9)	14 (76.5)		0.956
Appearance and body image	426 (64.3)	64 (92.3)	6.715 (2.659-16.960)	<0.001*	264 (53.7)	17 (94.1)	13.609 (1.791-103.435)	0.001*
Social interaction	143 (21.6)	12 (16.9)		0.380	151 (30.6)	7 (41.2)		0.352
Peer influence	34 (5.2)	3 (4.4)		0.461	62 (12.7)	3 (16.6)		0.911
Fun	438 (66.1)	26 (37.7)	0.327 (0.193-0.552)	<0.001*	381 (77.4)	12 (66.7)		0.216
Stress relief	560 (84.4)	57 (83.1)		0.788	360 (73.2)	17 (94.1)		0.053
Health benefits	527 (79.5)	53 (76.9)		0.626	333 (67.8)	12 (70.6)		0.807

Values are expressed as number of participants (percentage). * $p < 0.05$ indicates significant differences between participants at risk and participants with no risk, as determined by Pearson's chi-square ($\chi 2$). Odds ratios (ORs) with 95% confidence intervals were computed to estimate the magnitude of the significant associations (reference: non-ED risk).
ED: eating disorder.
